# Recent Developments of the Synthetic Biology Toolkit for *Clostridium*

**DOI:** 10.3389/fmicb.2018.00154

**Published:** 2018-02-12

**Authors:** Rochelle C. Joseph, Nancy M. Kim, Nicholas R. Sandoval

**Affiliations:** ^1^Department of Chemical and Biomolecular Engineering, Tulane University, New Orleans, LA, United States; ^2^Interdisciplinary Bioinnovation PhD Program, Tulane University, New Orleans, LA, United States

**Keywords:** clostridium, synthetic biology, CRISPR, metabolic engineering, biotechnology of microorganisms

## Abstract

The *Clostridium* genus is a large, diverse group consisting of Gram-positive, spore-forming, obligate anaerobic firmicutes. Among this group are historically notorious pathogens as well as several industrially relevant species with the ability to produce chemical commodities, particularly biofuels, from renewable biomass. Additionally, other species are studied for their potential use as therapeutics. Although metabolic engineering and synthetic biology have been instrumental in improving product tolerance, titer, yields, and feed stock consumption capabilities in several organisms, low transformation efficiencies and lack of synthetic biology tools and genetic parts make metabolic engineering within the *Clostridium* genus difficult. Progress has recently been made to overcome challenges associated with engineering various *Clostridium* spp. For example, developments in CRISPR tools in multiple species and strains allow greater capability to produce edits with greater precision, faster, and with higher efficiencies. In this mini-review, we will highlight these recent advances and compare them to established methods for genetic engineering in *Clostridium*. In addition, we discuss the current state and development of *Clostridium*-based promoters (constitutive and inducible) and reporters. Future progress in this area will enable more rapid development of strain engineering, which would allow for the industrial exploitation of *Clostridium* for several applications including bioproduction of several commodity products.

## Introduction

For production of fuels and chemicals, two competing design models exist. One design paradigm aims to endow some heterologous trait (e.g., a biomass utilization or production phenotype) onto a highly editable platform organism (e.g., *E. coli*) with the rationale that strain engineering can be performed more quickly and in a high-throughput manner. High throughput methodologies can enable rapid construction of balanced pathways (Smanski et al., 2014), synthetically designed genetic parts (Jones et al., 2015; Rohlhill et al., 2017), and whole-genome recoding (Ostrov et al., 2016). However, importing heterologous pathways often requires significant effort to achieve the production titers attained by native producing strains. The alternative approach is to improve strains which innately have the desired trait. Such strains already contain and use necessary genes and pathways, including cofactor regeneration. Engineering strains which already have a desired phenotype can avoid potential challenges such as the metabolic burden of high expression of heterologous genes, cofactor imbalance, genetic instability of imported genes or pathways, among others (Wu et al., 2016; Czajka et al., 2017; Wang M. et al., 2017). Development of an advanced genome engineering “tool kit” in non-platform organisms brings these two models closer together.

Advancements in the synthetic biology tool kit are required to better utilize the biotechnologically important capabilities of the *Clostridium* genus. The *Clostridium* genus is home to multiple industrially relevant strains. These Gram-positive, spore-forming, obligate anaerobic firmicutes are natively capable of cellulosic and hemicellulosic biomass degradation (e.g., *C. cellulolyticum*) (Heinze et al., 2017), carbon fixation (e.g., *C. carboxidivorans, C. ljungdahlii*) (Jones et al., 2016), advanced biofuel production (e.g., *C. acetobutylicum, C. beijerinckii*) (Liu K. et al., 2015), platform chemical production (e.g., *C. pasteurianum*) (Xin et al., 2016), and acting as anti-cancer therapeutics (*C. novyi-NT*) (Staedtke et al., 2016). Additionally, genome editing tools are of medical interest to better understand the many pathogenic strains in the genus (e.g., *C. botulinum, C. tetani, C. perfringens*) (Ng et al., 2013). *Clostridium* genome engineering has made much progress recently in the development of synthetic biology tools, although it still lags behind workhorse organisms (e.g., *E. coli*). Continued progress in this genus will enable broadened engineering on new platforms. In this mini-review, we discuss recent progress in the development of synthetic biology tools for members of the *Clostridium* genus and compare these to the established methods. Emphasized are CRISPR-based tools for genome editing and transcriptional perturbation as well as the library of genetic parts available for use in *Clostridium*.

## Gene editing

### ClosTron

#### ClosTron technology utilizes group II mobile introns for efficient targeted gene disruption in *Clostridium*

**Figure 1:**
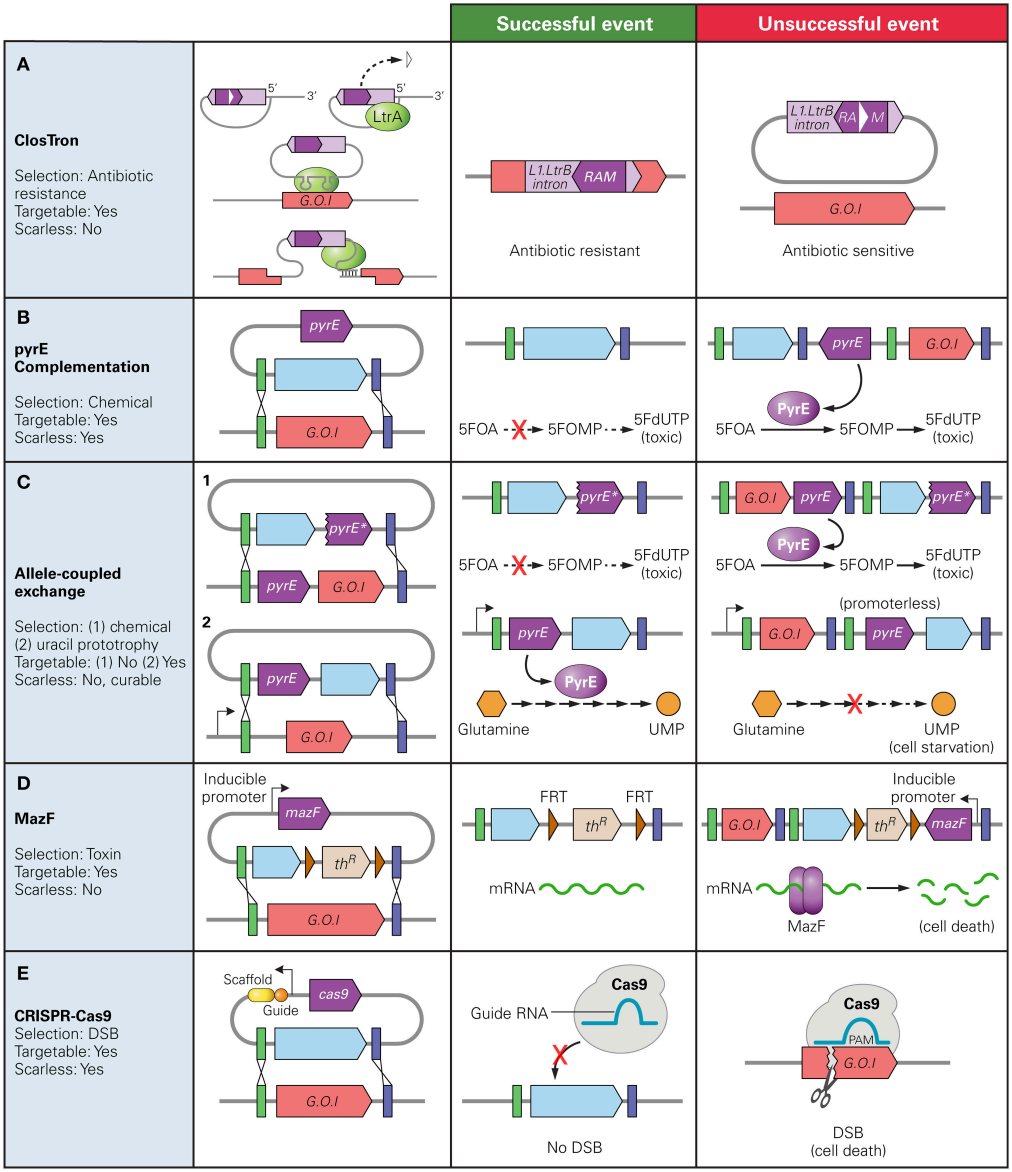
Counter Selection markers used in *Clostridium* spp. and their mechanisms of selection. The native gene of interest (G.O.I) is represented in red, desired insert in blue, and the counter selection marker gene in dark purple. The green and blue bars represent regions of homology between the chromosome and donor plasmid. **(A)** ClosTron: RAM disrupted by a Group I intron (white triangle) is only active after the L1.LtrB intron is inserted into the chromosome; **(B)** *pyrE* complementation: PyrE catalyzes conversion of 5-fluoroorotic acid (5FOA) to 5-fluororotidine monophosphate (5FOMP) producing toxic fluorodeoxyuridine monophosphate (FdUTP); **(C)** Allele-Coupled Exchange: (1) double-crossover event at the *pyrE* locus results in truncated version of pyrE for counter selection with same mechanism as **(B)**, (2) successful homologous recombination inserts a promoter-less copy of the *pyrE* gene directly downstream a native constitutive promoter, allowing production of uracil 5′ monophosphate (UMP). Note: must be performed on pyrE deficient strain; **(D)** MazF protein degrades mRNA at 5′-ACA-3′ sequences; **(E)** Cas9: successful homologous recombination gRNA-targeted double stranded break resulting in cell death.

Bacterial group II intron technology enables site-directed genetic disruptions based on the retrohoming of Mobile Group II introns. The mobility of Mobile Group II introns provides a convenient method of gene disruption as retrohoming is efficient and specific. The technology works by inserting an intron into chromosomal DNA through the plasmid-based monocistronic-expression of a ribonucleoprotein complex comprising RNA in a lariat configuration (acting as a ribozyme) and an intron-encoded protein (IEP). The Mobile Group II introns are minimally dependent on host factors, as the IEP (LtrA in the model system based on the *Lactococcus lactis* Ll.LtrB intron) performs multiple activities: maturase for facilitating RNA splicing, endonuclease for cleavage of the DNA strand opposite the RNA splice, and reverse transcriptase which uses intron RNA as template to insert DNA into the host chromosome. The host DNA repair machinery replaces intron RNA with DNA, completing the insertion. The term Targetron was first used to refer to targeted Group II introns when the L1.LtrB intron was further modified to include a retrotransposition-activated selection marker (RAM), providing a means to select for successful targeting events (Zhong et al., 2003). The RAM is inserted into the domain IV of the intron and consists a marker (often an antibiotic resistance gene) inactivated by the insertion of a Group I intron which is self-catalytically spliced out of mRNA in an orientation dependent manner. The marker gene and group I intron are oriented in the opposite directions such that it is only spliced out of the L1.LtrB mRNA, and a functional marker gene can only be expressed after successful chromosomal insertion occurs (Figure 1A).

The specific targeting, or retrohoming, is accomplished through protein-specific DNA binding as well as programmed RNA-DNA complementarity. This complementarity over a 13-nucleotide region allows for the specification of the DNA target site by altering the intron sequence. However, the target site is limited to DNA sites compatible with the IEP. The Targetron system has been used to perform gene targeting in both Gram positive and negative bacteria (Karberg et al., 2001) including several *Clostridium* spp.: *C. perfringens* (Chen et al., 2005, 2007), *C. acetobutylicum* (Shao et al., 2007) and *C. pasteurianum* (Pyne et al., 2014). ClosTron was developed as an adaptation of Targetron technology for efficient gene targeting specifically in *Clostridium* species. The original ClosTron plasmid, pMTL007, tailored the commercially available *E. coli* Targetron vector, pACD4k-C, to include standardized genetic parts such as promoters, origins of replications and RAMs suitable for efficient gene editing in *Clostridium* (Heap et al., 2007). ClosTron introns are designed using a computer algorithm which identifies suitable sequences, based on a 35 bp region recognized by the IEP, to which the intron can be targeted within the desired gene (Heap et al., 2010). These introns are expressed on vectors which follow the format of the pMTL80000 modular plasmids, allowing for the availability of a range of genetic parts. Flanking the RAM with FRT sites permits reuse of the same marker after FLP-FRT recombination (Heap et al., 2010). In *C. perfringens*, DNA fragments of up to 1.0 kb were successfully integrated into the chromosome in addition to an *ermB* RAM at a frequency of 5 × 10^−8^ integrants per cell, a 10^−4^ fold decrease in integration frequency when no additional cargo is added (Heap et al., 2010).

ClosTron has been employed in targeted gene disruption across the *Clostridium* genus including *C. acetobutylicum* (Hönicke et al., 2014; Liu Z. et al., 2015; Xu M. et al., 2015), *C. beijerinckii* (Heap et al., 2010; Liu et al., 2016), and *C. botulinum* (Meaney et al., 2015, 2016), as well as the closely related species, *Clostridioides difficile (Clostridium difficile*, Dingle et al., 2011; Baban et al., 2013; Hensbergen et al., 2015; Lawson et al., 2016). However, gene disruption using ClosTron is limited to IEP recognition sites, the frequency of which decreases as the length of the target sequence decreases. Additionally, while a scarless deletion is often preferred for gene knockouts, ClosTron merely disrupts the gene of interest and the intron, along with its RAM, remains in the host chromosome.

### Transposon-based random mutagenesis

#### Transposon mobile elements have been utilized in random mutagenesis in *Clostridium*

Among these, Tn916/Tn1545 family of transposons (called conjugative transposons) were among the first elements available for random gene insertions in *Clostridium*. These transposons consist of four functional modules responsible for conjugation, recombination, regulation and accessory functions (often antibiotic resistance). Although its expression can be plasmid-based, the conjugative ability of Tn916 (Flannagan and Clewell, 1991) has allowed its transfer from the chromosome of hosts such as *E. coli* and *Bacillus subtilis* into *Clostridium* species (Woolley et al., 1989). The use of Tn916-like transposons is limited by their large size, a predilection to insert in multiple copies or specific “hot spots,” and deletions at the site of insertion (Awad and Rood, 1997; Wang et al., 2000; Hussain et al., 2005). While non-conjugative transposon systems, such as Mu phage and EZ-Tn*5*-based transposon systems have addressed several of these concerns and have been successfully employed in *Clostridium* (Chen et al., 2005; Lanckriet et al., 2009; Vidal et al., 2009), they are limited by a preferential insertion into *rRNA* genes (Lanckriet et al., 2009; Vidal et al., 2009).

Mariner-transposable *Himar1*-based systems were developed for random mutagenesis in *Clostridium* (Cartman and Minton, 2010; Liu et al., 2013; Zhang Y. et al., 2015; Zhang et al., 2016). The *Himar1* transposable element, whose sequence includes a transposase gene flanked by short inverted terminal repeat sequences (ITRs), was originally discovered in the horn fly and has been shown to insert quasi-randomly into the genomes of several bacteria species, including members of the *Clostridium* genus. Successful *Himar1*-based events have been recorded in *C*. *acetobutylicum* (Zhang Y. et al., 2015), *C. perfringens* (Liu et al., 2013) and *C. sporogenes* (Zhang J. et al., 2015). *Himar1* transposase binds and cuts the element in its ITR region which, in turn, is inserted at a TA dinucleotide target site. This has been a valuable tool for mutagenesis in AT-rich *Clostridium* as it inserts average at one copy per cell.

### Counter-selection markers

#### Counter-selection markers enable the isolation of double crossover homologous recombination events in *Clostridium* spp.

Targeted gene disruption in bacteria can be achieved through plasmid-based, double crossover recombination events. The first event incorporates a vector containing a pair of homology arms flanking a cargo sequence into a target gene locus through homologous recombination (HR). In the second crossover, the specific region between the homologous sequences is deleted from the genome and is replaced by cargo DNA. Counter-selection markers are genetic elements which, when present, result in cell death; these markers are useful for selecting for chromosomal insertions which do not contain undesirable parts (e.g., the vector backbone of the recombination plasmid left over in a single-crossover event). Very few double-crossover events had been isolated within members of the *Clostridium* genus prior to the development of *Clostridium*-specific counter-selection marker systems (Awad et al., 1995; Bannam et al., 1995). Before routine usage of counter-selection markers, targeted homologous recombination in *Clostridium* had only been successful in a few species, the majority of which were segregationally unstable single cross-over integrations (Green and Bennett, 1996; Green et al., 1996; Nair et al., 1999; Liyanage et al., 2001; Harris et al., 2002; O'Connor et al., 2006). By contrast, the *sacB* gene from *B. subtilis* has been used to screen for double-crossover events in *E. coli* from the early 1990's.

One counter-selection marker method involves deactivating an easily screenable gene and then complementing the mutant strain with a heterologous version of that gene as a counter selective marker. Specifically, a disruption of *pyrE, pyrF*, or *upp* genes create uracil auxotrophic mutants which require supplementation for growth but are also resistant to the antimetabolites 5-fluoroorotic acid (5-FOA) or 5-fluorouracil (5-FU). By including a functional copy of the disrupted gene on the backbone of the donor DNA plasmid, double crossover events can be isolated as the mutants that demonstrate a resistance to 5-FOA or 5-FU (Tripathi et al., 2010; Heap et al., 2012; Croux et al., 2016) (Figure 1B). Similarly, in *C. perfringens*, disruption of the *galKT* operon produces mutants unable to produce the enzymes involved in galactose metabolism. GalK catalyzes the production of galactose-1-phosphate (Gal-1-P) from galactose, and GalT catalyzes its consumption. The accumulation of Gal-1-P is believed to inhibit cell growth by causing intracellular stress and inducing stress-responsive genes (Lee et al., 2009, 2014). By including only the *galK* gene and not the *galT gene* on the integration vector and plating mutant cells on galactose supplemented plates, unedited cells do not grow due to an accumulation of Gal-1-P while mutants that undergo a double-crossover event can be isolated (Nariya et al., 2011b).

Allelic Coupled Exchange (ACE) couples a counter selection marker gene to a desired double crossover event. This has been demonstrated in two ways. One method exploits the 5-FOA resistance conferred by a disrupted *pyrE* or *pyrF* gene. This method does not require the cells to be auxotrophic for uracil prior to recombination, nor does it rely on a heterologous version of the gene as a counter selection marker. ACE technology employs asymmetric homology arms to direct the order in which crossover events occur. The longer arm, homologous to a 1,200 bp region immediately downstream of the *pyrE* of *pyrF* directs the first crossover event in which the entire plasmid is incorporated into the genome. The second crossover event excises the plasmid backbone and is directed by the shorter arm which is homologous to a 300 bp internal region of the *pyrE* or *pyrF* gene. This second recombination replaces the wildtype *pyrE* gene with a truncated form thereby producing a mutant that can be screened based on 5-FOA resistance (Heap et al., 2012). Alternatively, a promoter-less heterologous *pyrE* gene or antibiotic marker can be inserted in the integration vector with the regions of homology such that a successful double crossover event places the silent gene directly downstream of a constitutive promoter (Heap et al., 2012). However, unlike previous methods which relied heavily on ClosTron technology to first produce auxotrophic mutants, *pyrE* mutants can be created utilizing ACE technology while the use of an antibiotic marker circumvents the need for a requisite mutant strain (Heap et al., 2012; Minton et al., 2016) (Figure 1C). ACE has been proven to be applicable over a range of *Clostridium* species, having been used for gene editing in *C. acetobutylicum* (Bankar et al., 2015; Ehsaan et al., 2016; Willson et al., 2016) and *C. sporogenes* (Heap et al., 2012; Zhang Y. et al., 2015), as well as *C. difficile* (Heap et al., 2012; Ng et al., 2013).

Several heterologous genes have been used for counterselection. The cytosine deaminase gene (*codA*) from *E. coli* can be used for counterselection based on the ability of the CodA protein to catalyze the conversion of 5-fluorocytosine (5-FC), an innocuous pyrimidine analog, to 5-FU (Cartman et al., 2012). *codA* can only be used for counterselection in strains with a functional *upp* gene but no native *codA* gene (Ehsaan et al., 2016). However, a bioinformatics survey suggests several *Clostridium* species contain *codA* homologs, restricting the applicability of *codA* among the genus (Al-Hinai et al., 2012).

Toxin-antitoxin systems are another useful source of counters-election markers. The *E. coli*-based *mazF* is an mRNA interferase, coded along with *mazE* in an operon. Under regular cell conditions, *mazE* binds to and inhibits *mazF* activity. During cellular stress, *mazE* is degraded, allowing *mazF* to bind mRNA, degrading them at 5′-ACA′3′ sequences, thereby arresting cell growth. *mazF*, coupled with an antibiotic resistant marker flanked by FRT sites, can be used as a counter selection marker in plasmid based homologous recombination. *mazF* is placed on the gene disruption plasmid under the control of an inducible *lac* promoter. A double crossover event can be isolated in cells able to grow on lactose-supplemented plates (Al-Hinai et al., 2012) (Figure 1D). The use of *mazF* requires no prior mutation for successful screening, is independent of the availability of *Clostridium* genetic parts, and has been shown to function across *Clostridium* species (Al-Hinai et al., 2012; Sandoval et al., 2015; Zhang J. et al., 2015). Flp-frt recombination has also been used to eliminate the backbone of a donor plasmid following a single-crossover event in *C. acetobutylicum*, allowing for the use of an antibiotic gene as a marker for the crossover event after the donor plasmid had been cured (Lee S. H. et al., 2016).

### Crispr-based editing

#### CRISPR/Cas9 allows efficient, marker-less gene editing in *Clostridium*

Clustered Regularly Interspaced Palindromic Repeats (CRISPR), along with its CRISPR associated (Cas) proteins is an adaptive immunity system in prokaryotes (Barrangou et al., 2007; Wiedenheft et al., 2012). The type II CRISPR system native to the *Streptococcus pyogenes* bacterium was the first CRISPR system exploited for gene engineering (*spy*Cas9) (Jinek et al., 2012). This CRISPR system consists of the single Cas9 effector protein, which can bind to and implement a double stranded break (DSB) to a targeted DNA system when co-expressed with a single guide RNA targeting a 20 bp region immediately adjacent to the protospacer adjacent to a motif (PAM). In the case of *spy*Cas9, the PAM consensus sequence is NGG, providing many possible target sites.

The CRISPR/Cas9 system has been used as a counter-selection tool to select for homologous recombination events in several *Clostridium* species (Wang et al., 2015; Bruder et al., 2016; Huang et al., 2016; Nagaraju et al., 2016; Wang S. et al., 2017; Wasels et al., 2017) (Table 1). *Clostridium* spp. lack or have inefficient non-homologous end-joining (NHEJ) systems, so a Cas9-mediated chromosomal DSB results in cell death (Cui and Bikard, 2016; Xu et al., 2017). Thus, to select for successful homologous recombination events, one can selectively eliminate non-edited members of the population by targeting the wild type sequence (Figure 1E). Studies in *E. coli* have shown the DSB can enhance homologous recombination in bacteria, whereby homology directed repair (HDR) occurs after a break has been induced (Jiang et al., 2013). However, studies in different *Clostridium* species suggest that HDR efficiency in these species is too low to select for successful HDR events (Wang et al., 2015, 2016a; Li Q. et al., 2016). The use of CRISPR/Cas9 represents a major advancement in *Clostridium* gene editing in *Clostridium* as scarless edits are enabled.

**Table 1:** CRISPR-based genetic editing and gene repression in *Clostridium* spp.

**Effector**	**Species**	**Homology arm length (bp)**	**Transformation Eff. (CFU/μg)**	**Editing efficiency (%)**	**Cas9 promoter**	**Gene targeted**	**Desired edit**	**Citation**
Cas9	*C. acetobutylicum*	664	NR	100	*tet* (inducible)	*upp*	DNM	Wasels et al., 2017
		500	NR	100		*upp*	66 bp del	
		1,000	NR	100		*upp*	306 bp rep	
	*C. beijerinckii*	1,000	NR	67	*spoIIE*	*pta*	50 bp del	Wang et al., 2016a
		1,000	1.05 ^*^ 10^2^	80	*bgaL* (inducible)	*pta*	50 bp del	
		1,000	3.94 ^*^ 10^2^	0		*pta*	1,500 bp del	
		1,000	2.92 ^*^ 10^2^	87		*pta*	1,614 bp ins	
		1,000	*NR*	>99		*pta*	SNM	
		1,000	*NR*	NR	*spoIIE*	*spo0A*	262 bp del	Wang et al., 2015
	*C. autoethanogenum*	*NR*	*NR*	>50	*tet* (inducible)	*caethg_ 0385*	del	Nagaraju et al., 2016
		*NR*	*NR*	>50		*caethg_ 05552*	del	
	*C. acetobutylicum*	500	0.2	100	*thl*	*cac1502*	rep w/trunc. gene	Bruder et al., 2016
		1,000	0.38	100		*cac1502*	rep w/trunc gene	
		1,000	0.4	NR		*cac1502*	rep w/ Pthl::afp	
	*C. Ijungdahlii*	*NR*	*NR*	100	*ptb*	*pta*	1,000 bp del	Huang et al., 2016
		*NR*	*NR*	>75		*adhE1*	2,600 bp del	
		*NR*	*NR*	100		*ctf*	1,200 bp del	
		*NR*	*NR*	>50		*pyrE*	570 bp del	
	*C.saccharoperbutyl-acetonicum N1-4*	1,000	1.5 ^*^ 10^4^	NR	*bgaL* (inducible)	*buk*	del	Wang S. et al., 2017
		1,000	1.6 ^*^ 10^4^	75		*pta*	del	
	*C. pasteurianium*	1,000	2.6	100	*thl*	*cpaAIR*	567 bp del	Pyne et al., 2016
Cas3^*^	*C. pasteurianium*	*NR*	9.5	100	N/A	*cpaAIR*	762 bp del	Pyne et al., 2016
Cas9n	*C. cellulolyticum*	1,000	*NR*	100	*fdx*	*pyrF*	23 bp del	Xu T. et al., 2015
		1,000	*NR*	NR		*mspI*	23 bp del	
		500	*NR*	100		*X-21*	12 bp del	
		200	*NR*	100		*X-22*	12 bp del	
		100	*NR*	<95		*B-gal*	6 bp ins	
		200	*NR*	<95		*B-gal*	6 bp ins	
		500	*NR*	>95		*B-gal*	6 bp ins	
		1,000	*NR*	>95		*B-gal*	6 bp ins	
		1,000	*NR*	100		NR	710 bp ins	
		1,000	*NR*	100		NR	1,720 bp ins	
		1,000	*NR*	0		NR	3,000 bp ins	
		1,000	*NR*	0		NR	6,000 bp ins	
	*C. acetobutylicum*	*NR*	15.5	30	*ptb*	*pyrE*	20 bp del	Li Q. et al., 2016
		*NR*	*NR*	7		*adc*	20 bp del	
		*NR*	*NR*	100	*thl*	*agrA*	20 bp del	
	*C. beijerinckii*	*NR*	*NR*	19	*thl*	*adc*	20 bp del	
		*NR*	14.6	98		*xlyR*	20 bp del	
		150	*NR*	0		*xlyR*	20 bp del	
		200	*NR*	0		*xlyR*	20 bp del	
		500	*NR*	30		*xlyR*	20 bp del	
		1,000	*NR*	100		*xlyR*	20 bp del	
		*NR*	*NR*	50		*araR*	20 bp del	
		*NR*	*NR*	100		*cbei3923*	20 bp del	
		*NR*	*NR*	40		*cbei4495*	20 bp del	
		*NR*	*NR*	43		*xylR*	1149 bp del	
	*C. cellulolyticum*	*NR*	*NR*	NR	NR	Ccel_3198^**^	120 bp ins	Xu et al., 2017
**Type**	**Species**	**Target strand**	**Repression (%)**	**dCas9 expression**		**Gene targeted**		**Citation**
dCas9	*C. beinjerinckii*	*NR*	65–95	*thl*		*amy*		Wang et al., 2016b
	*C. acetobutylicum*	*NR*	45	*ptb*		*spo0A*		Li Q. et al., 2016
	*C. beinjerinckii*	*NR*	84			*spo0A*		
	*C. celluvorans*	*NR*	95	*thl*		*nuoG*		Wen et al., 2017
	*C. acetobutylicum*	Nontemplate	90	*ptb*		Plasmid-based *afp*		Bruder et al., 2016
		Template	20			Plasmid-based *afp*		
		Nontemplate	NR			*hprK*		
		Nontemplate	NR			*glpX*		

All mentions of Cas9 refer to Streptococcus pyogenes-derived Cas9. Reported editing efficiencies are the fraction of successful mutants of total colonies screened. del, deletion; DNM, dinucleotide modification; ins, insertion; NR, Not reported; rep, replacement; SNM, Single nucleotide modification; trunc, truncation.

* Cas3 is the effector protein in the native C. pasteurianum type I-B CRISPR system.

** *Targeted region downstream of Ccel_3198 gene*.

The limited number of characterized genetic parts for *Clostridium* poses a challenge with CRISPR/Cas9 engineering. For example, simultaneous constitutive expression of the sgRNA and Cas9 protein often resulted in few to no transformed colonies in the presence of a homologous repair donor vector, as DSBs result in cell death before recombination can occur (Bruder et al., 2016; Li Q. et al., 2016; Nagaraju et al., 2016; Wang et al., 2016a). This can be addressed by placing Cas9 expression under the control of an inducible promoter (Nagaraju et al., 2016; Wang et al., 2016a; Wang S. et al., 2017; Wasels et al., 2017). Another strategy is to use a two-plasmid system, where the donor DNA and sgRNA are introduced separately from the Cas9 gene. This method avoids the transformation of very large plasmids, which have reduced transformation efficiency, but it requires two separate transformation events (Wasels et al., 2017). Using these methods, successful recombinants were isolated at a rate up to 100% (Wasels et al., 2017) with commonly observed efficiencies of greater than 50% (Table 1).

Cas9 nickase (Cas9n) systems exploit CRISPR gene editing while circumventing the lethality associated with the co-expression of a guide RNA with Cas9. This method utilizes Cas9n, a mutated form of the Cas9 protein, with the ability to only cut one DNA strand. While simultaneous expression of Cas9 and guide RNA is fatal to cells in the absence and presence of a donor template, implementing a single nick into the genome via Cas9n allowed homologous recombination without the lethal effects of Cas9, thus permitting a mixed population of edited and unedited strains to coexist. (Xu T. et al., 2015) Therefore, several serial dilutions are required to enhance the edited population through increased likelihood of homologous recombination at the nicked site, reduced growth rate of the nicked strains, or some combination of both. CRISPRn has been used to implement gene deletions and insertions in *C. acetobutylicum, C. beijerinckii* and *C. cellulolyticum* with up to 100% efficiency (Xu T. et al., 2015; Li Q. et al., 2016; Xu et al., 2017) (Table 1).

Longer regions of homology on the donor have been shown to increase efficiency. In *C. cellulolyticum*, donor template arm lengths greater than 0.2 kb had an efficiency of more than 95% when compared with smaller arms which were only 55% efficient in a CRISPRn system (Xu T. et al., 2015). A similar study using Cas9 in *C. acetobutylicum* demonstrated an increased efficiency when homology arm lengths of 1 kb were used as opposed to 500 bp arm lengths (Bruder et al., 2016).

Application of these advanced CRISPR tools is still limited in *Clostridium* due to low plasmid transformation efficiencies and a lack of characterized recombineering and NHEJ tools. Recombineering, through lambda red technology, has facilitated gene engineering in *E. coli* via the use of linear DNA repair templates, a process that skips the cloning steps required in plasmid-based homologous recombination methods. Coupled with CRISPR, this technology enables multiplexed ssDNA recombineering events with efficiencies allowing large libraries (>10^5^ members) to be constructed in parallel (Ronda et al., 2016; Garst et al., 2017). However, the lack of ssDNA recombineering machinery functional in *Clostridium* hinders the development of comparable *Clostridium*-based technologies. Although a RecT protein from *C. perfringens* demonstrated recombineering activity in *C. acetobutylicum* recently, the results obtained were not comparable to routine recombineering events in *E. coli* (Dong et al., 2014). Similarly, the expression of Ku and LigD genes from *Mycobacterium tuberculosis* enabled NHEJ in *E. coli* following the implementation of a DSB via Cas9 (Su et al., 2016). Although NHEJ related genes (*ku, DNA ligase*, and *ligD*) are found on the *C*. *cellulolyticum*, they are not highly expressed and NHEJ events have not been observed in the species after a Cas9 DSB (Xu T. et al., 2015). Heterologous expression of such genes may enable NHEJ in *Clostridium*.

Repurposing endogenous CRISPR systems has been proposed as an alternative to transforming and expressing CRISPR-Cas genes in *Clostridium*. A type I-B CRISPR system was identified in *C. pasteurianum* based on its genome sequence. The PAM sequence recognized by its Cas effector protein was determined via a bioinformatics survey of existing spacer sequences This system was used to target the *cpaAIR* gene in *C. pasteurianum* when transformed with a plasmid based expression copy of its CRISPR array and a homology repair template (Table 1). The donor DNA/targeting plasmid was transformed at a higher efficiency compared to a similar Cas9 based system, since the plasmid did not need to house the large CRISPR effector protein (9.5 vs. 2.6 CFU/μg) (Pyne et al., 2016). While the use of native CRISPR systems circumvents the need to express heterologous Cas effector proteins, the applicability of these systems may be limited to strains with functional CRISPR/Cas machinery and by unknown PAM sequences. Although application has not been reported in *Clostridium*, other CRISPR-Cas9 systems have been identified in other bacteria, including thermophilic Cas9 systems which can be utilized for gene editing in thermophiles (Mougiakos et al., 2017b) in lieu of *spy*Cas9 as it is temperature sensitive (Mougiakos et al., 2017a).

#### Gene silencing in *Clostridium* can be achieved via a catalytically dead Cas9 mutant

The catalytically dead Cas9 (dCas9) does not demonstrate endonuclease activity but retains its ability to bind to DNA at a specified target region. In a CRISPR interference (CRISPRi) system, dCas9 is targeted to a sequence by a guide RNA and binds to it, sterically hindering transcription initiation. CRISPRi has enabled simple, tunable, reversible gene knockdown at a transcriptional level. One needs only to express sgRNAs and with the aid of bioinformatics tools, can specifically implement gene expression knock-downs. This method is reversible, with no permanent change in the genome (Qi et al., 2013). Additionally, CRISPRi activity can be modulated not only through controlling the expression of dCas9 (Li X. T. et al., 2016), but also by the relative position of the dCas9 protein to the promoter and gene start site (Kim et al., 2017), allowing tight control of gene expression. CRISPRi technology has been used for gene knockdowns in *C. acetobutylicum* (Bruder et al., 2016; Li Q. et al., 2016), *C. beijerinckii* (Li Q. et al., 2016; Wang et al., 2016b) and in *C. cellulovorans* (Wen et al., 2017) and has been used to silence both native (Li Q. et al., 2016; Wang et al., 2016b; Wen et al., 2017) and heterologous genes (Bruder et al., 2016) (Table 1). Gene repression of up to 97% was achieved although the effectiveness of CRISPRi varies among species with similar configurations (Li Q. et al., 2016). The tunability of dCas9 has yet to be fully explored in *Clostridium*, as it has been in other bacterial systems (Li X. T. et al., 2016; Kim et al., 2017). In fact, one study showed activity of the knockdown target unintentionally increased over the time of fermentation (Wang et al., 2016b). This increase was attributed to variable strength of the thiolase promoter, which controlled the transcription of dCas9.

Gene expression knockdowns have also been accomplished on the translational level in *Clostridium* through antisense RNA (asRNA) technology. The asRNA knockdown method involves targeting an mRNA transcript using its asRNA. This method has been used to investigate the function of genes in various *Clostridium* species (Cooksley et al., 2010; Fagan and Fairweather, 2011; Chandrasekaran et al., 2016; Chu et al., 2016; Xu et al., 2017), several of which are essential genes, making a genetic knockout unfeasible. asRNA technology has also been used to manipulate gene expression affecting solvent titers (Tummala et al., 2003; Sillers et al., 2009). asRNAs are tunable and reversible, and have been effective in silencing, achieving as high as 90% repression levels of certain genes. However, despite their lengths (>100 bp) asRNAs have been shown to be promiscuous, binding especially to transcripts with a high homology to the target sequence (Cooksley et al., 2010). Consequently, asRNA technology requires large constructs for efficient gene repression while CRISPRi provides repression specificity, using a 20 nt sgRNA choice.

## Genetic parts

In addition to development of DNA editing tools, a “toolbox” of well-characterized biological parts including promoters, ribosomal binding sites (RBS), origin of replication (ORI), and terminators for *Clostridium* is in need for further advancement of metabolic engineering efforts. Expanding this toolbox would enable assemblies of individual or grouped genes to enhance productivity and yield of desired products or outcomes.

### Promoters

Promoters are often the simplest way to control transcription, and promoters with various activities are valuable in any synthetic biology toolkit. However, promoters available for use in *Clostridium* originate from a few strains and are not always transferrable to non-native hosts. Constitutive promoters of *ptb* (phosphotransbutyrylase) and *thl* (thiolase) from *C. acetobutylicum* and *C. pasteurianum* are commonly used, however, promoter activity vary in different strains and stages of growth (Pyne et al., 2015; Lee J. et al., 2016; Yang et al., 2017). Therefore, there is a need for well-characterized promoter parts in synthetic biology.

Whole-transcriptomic sequencing offers comprehensive profiles of gene expression and transcriptome changes of an organism with high precision. Promoter motifs identified from RNA-seq analysis of *E. coli* and gram-positive *B. subtilis* are commonly used to screen for promoters in other organisms using bioinformatics tools (Borden et al., 2010). Promoter motifs are less well-established in *Clostridium*, thus relying on promoter motifs of *B. subtilis* as a reference (Paredes et al., 2004). Successful promoter prediction through bioinformatics requires more transcriptomic studies in *Clostridium*.

As screening for native promoters is time-consuming, mutagenesis methods such as error prone PCR, saturation mutagenesis, or site-specific mutagenesis of a known characteristic such as a hairpin are utilized to rapidly generate a library of synthetic promoters with varying strengths. A library of synthetic promoters using a web-based tool WEBLOGO was used to rapidly generate a library of promoters of various strengths by randomizing the flanking regions surrounding the -35 and -10 consensus sequences of promoter *thl* for *C. acetobutylicum* and *C. ljungdahlii* (Yang et al., 2017).

Several inducible promoters have been used to balance and map metabolic pathways, study function and regulation of promoters or genes, and express recombinant proteins in *Clostridium*: xylose-inducible promoter-repressor system from *Staphylococcus xylosus* (Girbal et al., 2003; Nariya et al., 2011a); lactose-inducible promoter comprising of a transcriptional regulator gene, *bgaR*, and a *bgaL* promoter from *C. perfringens* (Hartman et al., 2011; Al-Hinai et al., 2012; Banerjee et al., 2014); laminaribiose-inducible promoters (Mearls et al., 2015); arabinose-induced promoter (Zhang J. et al., 2015); tetracycline-inducible system (Walker and Köpke, 2015), anhydrotetracycline-inducible system (Dong et al., 2012); and radiation-induced promoter in *C. acetobutylicum* for cancer radiation therapy (Nuyts et al., 2001). Factors to be cognizant in using inducible promoters are its sensitivity to the inducer, as the cost of inducers may increase for large-scale production applications, and “leaky” expression in the absence of the inducer. Therefore, a quantitative measurement of transcription is necessary for characterization of promoters.

### Reporters

Genetic reporters, genes which act as an observable proxy for some unobservable process, can provide measurement of gene expression and screening and characterization of promoters. An ideal reporter system has (1) high sensitivity and specificity, (2) a large dynamic range of detection, and (3) low endogenous levels of the reporter in the strain of interest. Chloramphenicol acetyltransferase (*catP*) has been used to select for active promoters by catalyzing the transfer reaction of an acetyl group from acetyl-CoA to the antibiotics chloramphenicol (Cm) and thiamphenicol (Bullifent et al., 1995). While Cm is effective in *C. perfringens*, other species such as *C. acetobutylicum* and *C. beijerinckii* NCIMB 8052 are naturally resistant to the antibiotic (Feustel et al., 2004). As the reporter mechanism is selection, it is not ideal for measurement of specific promoter activity. Interestingly, a *catP*-*lacZ* fusion reporter provides an initial screen to select for *catP* expressing promoters on agar with varying Cm concentrations, and subsequent colorimetric screen to measure accurate promoter activities (Yang et al., 2017).

*lacZ* from *Thermoanaerobacterium thermosulfurigens* EM1, as well as *gusA* (or *uidA*) from *E. coli* encoding β-galactosidase and β-glucuronidase respectively, are used to study quantitative and qualitative gene expression in *Clostridium* (Tummala et al., 1999; Feustel et al., 2004). A major advantage of the *lacZ* and *gusA* genes is the versatility of detection systems, depending on the substrate. *lacZ* reporter substrates reported in *Clostridium* include the substrates 5-bromo-4-chloro-3-indolyl-β-*D*-galactoside (X-GAL) (Feustel et al., 2004), *o*-nitrophenyl-β-*D*-galactopyranoside (ONPG) for colorimetric and spectrophotometric assays (Adcock and Saint, 2001; Tan et al., 2015), and 4-methylumbelliferyl-β-*D*-galactopyransoside (MUG) for fluorescent assays (Adcock and Saint, 2001). *gusA* reporters utilize analogous substrates to β-galactosidase assays: MUG for fluorescence, 5-bromo-4-chloro-3-indolyl-glucuronide for colorimetric, and *p*-nitrophenyl-β-*D*-glucuronide for spectrophotometric detection. Compared to *lacZ, gusA* is smaller and more stable with low background as a fusion reporter with the gene of interest in many organisms including *Clostridium* spp. (Ravagnani et al., 2000; Mani et al., 2006). Although not commonly used, other enzymatic-based reporters available include the alkaline phosphatase gene (*phoZ*) isolated from *Enterococcus faecalis* (Gibson and Caparon, 2002) and the β-1,4-endoglucanase gene (*eglA*) from *C. saccharobutylicum* (Quixley and Reid, 2000).

Bioluminescent reporters provide amplified measurements of genes expressed at low quantities. Despite the need for oxygen, luciferase reporters (*luc* and *lucB*) from North American firefly (*Photinus pyralis*) in *Clostridium* spp. can be achieved with the addition of ATP and washing cells with a neutral pH buffer (e.g., PBS) (Davis et al., 2000; Phillips-Jones, 2000; Feustel et al., 2004). In addition, luciferase fusion with secreting protein PPEP-1 in *C. difficile* enabled low-background luciferase expression in an anaerobic environment and luminescence measurement in an aerobic environment, albeit with a delayed signal (Oliveira Paiva et al., 2016). Likewise, bacterial luciferases (*luxAB*) of the lux system (*luxCDABE*) from *Vibrio fischeri* provide a luminescent output in the presence of flavin mononucleotide and long-chain aldehyde substrate in *C. perfringens* (Bullifent et al., 1995; Phillips-Jones, 2000). Although various strategies enable use of bioluminescent reporters in anaerobic bacteria, transcription activity is not directly correlated with the output (Iqbal et al., 2017).

Fluorescent reporters such as green fluorescent protein (GFP) and its variants allow real-time measurements of gene expression at a single cell level without the need of substrates. Although GFP variants CFP and mCherryOpt can be expressed in *Clostridium*, exposure to oxygen is required for the intrinsic chromophore to fluoresce (Ransom et al., 2015). FMN-based fluorescent proteins (FbFPs) including LOV (Light Oxygen Voltage) domains from plants and bacterial blue light receptors are an emerging class of fluorescent reporters for anaerobes (Drepper et al., 2007; Christie et al., 2012; Seo et al., 2018). These fluorescent reporters can be utilized with and without oxygen, light, and voltage, hence the name. They are smaller (~13 kDa) than GFP (~25 kDa) allowing relatively faster turnover rates and fusion with protein of interest without disrupting its native function. Furthermore, improved LOV (iLOV) proteins can retain 60–70% fluorescence in a broad pH range of 4–11 (Mukherjee et al., 2013). Excitation of improved LOV (iLOV) with blue-light wavelength at 450 nm emits an emission peak at 495 nm (Mukherjee et al., 2013), while GFP excitation and emission spectra are at 488 and 507 nm, respectively. iLov has been utilized as a fluorescent reporter in several *Clostridium* species: *C. cellulolyticum* (Teng et al., 2015), *C. ljungdahii* (Molitor et al., 2016), as well as *C. difficile* in which it was used for real-time measurement of protein localization and secretion via fusion to FtsZ, a cell division protein (Ransom et al., 2015; Teng et al., 2015; Buckley et al., 2016; Molitor et al., 2016). iLov expression has also been observed in *C. acetobutylicum* (Buckley et al., 2016).

The quantum yield (brightness) of iLOV is lower than GFP, however. Thus, there are some considerations to lower noise when using FbFPs. Flavin-based media such as reinforced clostridial medium (RCM) and PETC give high fluorescent backgrounds which can be reduced by eliminating yeast and beef extract (Molitor et al., 2016). Also, species such as *C. acetabutylicum* have a natural green auto-fluorescence that can increase the background signal (Buckley et al., 2016). A negative control plasmid containing a non-fluorescent reporter such as *gusA* can be utilized to tune excitation wavelength (i.e., 450 vs., 470 nm) to improve noise-to-signal fluorescence (Buckley et al., 2016).

### Origins of replication

As the plasmid is a fundamental synthetic biology tool, the origin of replication is fundamental to successful plasmid stability. Several origins of replication have been found to be effective in *Clostridium* species including: pBP1, pCB102, pCD6 and pIM13, which are included in the pMTL80000 series plasmids (Heap et al., 2009). Origins pBP1 and pCB102 are native to *Clostridium* (*C. botulinum* and *C. butyricum*, respectively), while pIM13 and pCD6 were isolated from *B. subtilis* and *Clostridioides difficile*, respectively. The origin-strain pair exhibit varying levels of stabilities. For example, pCB102 is stable in *C. botulinum* ATCC 305, but not as stable in *C. acetobutylicum* 824 (Minton et al., 2016).

Conditional origins are useful for when the presence of a plasmid is required only under certain conditions, such as recursive recombination editing. In *C. acetobutylicum*, the pAMβ1 replicon from *E. faecalis*, is unstable in the absence of antibiotic challenge (Lee S. H. et al., 2016). Recently, a temperature sensitive origin of replication derived from the pWV01 from *Lactococcus lactis* subsp. *cremoris* was tested in *C. ljungdahlii*, having the plasmid maintained at a permissive temperature of 20°C but lost by dilution at 37°C (Molitor et al., 2016).

### Terminators

Terminators play an important role in gene expression stability. Often overlooked, transcriptomic studies and computational tools such as TransTerm and RNAMotif can identify and predict *Clostridium* terminators (Chen et al., 2011; Wang et al., 2011). The *adc* (*acetoacetate decarboxylase*) terminator from *C. acetobutylicum* and *fdx* (ferrodoxin) terminator from *C. pasteurianum* (Cartman and Minton, 2010; Fagan and Fairweather, 2011; Nariya et al., 2011b; Pyne et al., 2015; Zhang Y. et al., 2015) are the most commonly used. The terminator of the *C. difficile* 630 ferrodoxin gene, CD0164, was identified *in silico* and is present on the pMTL80000 plasmids routinely used in several *Clostridium* species (Heap et al., 2009; Ng et al., 2013). Non-native terminators, including bidirectional *E. coli* terminator BB1_B1010 from iGEM Parts Registry, have demonstrated stronger termination over native *adc* terminator (Lee J. et al., 2016).

## Conclusions

Several *Clostridium* spp. have the potential to be platform organisms for industrial use. However, additions to the available toolkit are required for high-throughput methods of strain engineering. One consistent hurdle is low DNA transformation efficiency, commonly as low as 10 CFU/μg (Table 1). However recent efforts to improve transformation protocols (Pyne et al., 2013) and the isolation of a hyper-transformable strain have resulted in higher efficiencies (Grosse-Honebrink et al., 2017; Schwarz et al., 2017). Further directions include library-based methods requiring high transformation efficiencies which would prove to be a step change improvement over current screening method. Although several studies have focused on increasing the availability of parts such as promoters and reporters, the synthetic biology toolkit can further be expanded through the characterization of other genetic parts such as ribosomal binding sites and terminators. Improvement in these areas will accelerate progress toward a sustainable bio-based economy.

## Author contributions

RJ, NK, and NS prepared and edited the manuscript; RJ prepared figure and table.

### Conflict of interest statement

The authors declare that the research was conducted in the absence of any commercial or financial relationships that could be construed as a potential conflict of interest.
